# Disposable aptamer-sensor aided by magnetic nanoparticle enrichment for detection of salivary cortisol variations in obstructive sleep apnea patients

**DOI:** 10.1038/s41598-017-17835-8

**Published:** 2017-12-21

**Authors:** Renny Edwin Fernandez, Yogeswaran Umasankar, Pandiaraj Manickam, Jeffrey C. Nickel, Laura R. Iwasaki, Burt K. Kawamoto, Kristen C. Todoki, JoAnna M. Scott, Shekhar Bhansali

**Affiliations:** 1 Bio-MEMS and Microsystems Laboratory, Department of Electrical and Computer Engineering, Florida International University, Miami, Florida USA; 2 Biomolecular Sciences Institute, Florida International University, Miami, Florida USA; 3 School of Dentistry, Departments of Orthodontics and Dentofacial Orthopedics and Oral and Craniofacial Sciences, University of Missouri-Kansas City, Kansas City, Missouri USA; 4 School of Dentistry, University of Missouri-Kansas City, Kansas City, Missouri USA; 5 School of Dentistry, Department of Oral and Craniofacial Sciences, University of Missouri-Kansas City, Kansas City, Missouri USA

## Abstract

We report a disposable point-of-care sensing platform specific to salivary cortisol detection. The sensor is inkjet printed on a paper substrate with a metalloporphyrin based macrocyclic catalyst ink that can electrochemically reduce cortisol, captured by aptamer functionalized magnetic nanoparticles. The sensor consists of a thin magnet disc, aligned at the back of the electrode, in order to populate the magnetic nanoparticle bound cortisol at the sensing electrode area. Proof of concept studies were performed to detect salivary cortisol levels in human subjects with high and low risks for obstructive sleep apnea (OSA). High selectivity was observed to salivary cortisol against a background of closely related steroids.

## Introduction

Scientific community has always shown great interest in inexpensive devices and sensors that can detect biomolecular levels from human body fluids. There has been a lot of recent interest in low-cost flexible electronics^1,2^, enabled by innovative materials and printing technologies^3,4^. Low-cost mass-production processes are the need of the hour to meet the requirement of point-of-care sensing. Disposable electrodes are traditionally realized via screen printing^5,6^, a low-cost technology. However, the screen printing process requires adequate masks which also limits its resolution. Due to considerable wastage involved in screen printing, the total cost of such a process is high^7^. In comparison, inkjet printing has obvious advantages. Flexible substrates and functional inks have been used along with inkjet printing^8,9^. They are also extremely suitable for rapid prototyping where a designer can instantly realize and test a device, which shortens the design and testing cycle. Conductive metal inks, resistive polymers, e.g., poly 3,4-ethylenedioxythiophene polystyrene sulfonate (PEDOT-PSS), and functional polymers, e.g., polianiline-PANI, are widely used for the realization of sensors via inkjet printing^10,11^. Various redox enzymes and proteins are capable of electrocatalyzing reactions via direct electron transfer. Despite their redox capability, proteins and most biomolecules are highly sensitive to denaturing. Alternatively, an aptamer is highly stable and not susceptible to denaturing.

The most promising approach for the development of electrochemical biosensors is to establish a direct electrical communication between the biomolecules and the electrode surface. We introduce a conjugate of multi-walled carbon nanotubules (MWNT) and Metalloporphyrin based ink that can catalyze the reduction of cortisol, at the working electrode of our sensor. Metalloporphyrins are a crucial part of organic macrocyclic catalysts that are widely distributed in nature, playing essential roles in vital processes like photosynthesis and oxygenation^12,13^. They also can act as enzyme mimics, capable of catalyzing a reaction such as cortisol reduction. For instance: 11-β-hydroxysteroid dehydrogenase 2 is an enzyme that catalyses the reduction of cortisol to cortisone^14,15^. Organic macrocyclic catalysts that can act as enzyme mimics and imitate this catalysis reaction can be designed by conjugating transition metal elements such as Re, Ru, Co, Ni with macrocyclic structures like porphyrin for electrochemical reduction/oxidation of various biomolecules^16–18^. These enzyme mimics can attain the kinds of regioselectivity otherwise possible only with enzymes. Many studies have reviewed the epoxidation and hydroxylation of steroid derivatives using *tert*-butyl hydroperoxide (TBHP), 2,6-dichloropyridine *N*-oxide, iodosylbenzene or H_2_O_2_ as oxygen donors in the presence of Cu, Fe, Ru, Mn, or Os porphyrins^14,19^.

Our sensor uses a cortisol specific aptamer to attain high specificity to cortisol and to counter non-specific adsorption of similar steroids. Aptamers are fast replacing antibodies in affinity based biosensing. They can be covalently immobilized on most surfaces by modifying the 5′ or 3′ end. Aptamers are highly selective and less sensitive to temperature fluctuations of the medium. However, aptamers alone cannot function as biosensing units and must be conjugated with a sensing strategy that can utilize the selectivity of the aptamers. Electrochemical techniques that rely on the electrode resistance changes as a result of aptamer-analyte binding have been reported^20,21^. However, the magnitude of decrease in peak currents due to affinity binding can generate false negatives because these reflect increments in electrode resistance due to target-probe binding which do not involve electrochemical reactions. Hence, an affinity biosensor is best served in combination with an electrochemical reaction. We have used aptamer functionalized magnetic nanoparticles to ensure selectivity of our sensing platform. Magnetic nanoparticles, functionalized with affinity molecules, can enable rapid capture of a specific target molecule from a heterogeneous environment. Coupling of electrochemical transduction with magnetic nanocarriers has greatly improved the sensitivity of electrochemical biosensors^22,23^. We have enabled our disposable device with magnetic enhancement by including a magnetic strip, aligned at the back of the working electrode. Typically, magnetic nanoparticles are immobilized with a capture molecule that has an affinity for a specific target. Hence, magnetic nanoparticles introduced into the sensing area are confined to the working electrode.

Herein, we propose a disposable platform for salivary cortisol detection which is cost effective, selective and rapid. The inkjet printed platform with a metalloporphyrin modified working electrode is used in conjunction with aptamer immobilized nanoparticles in a magnet backed disposable sensor. The sensing platform was used to detect the salivary cortisol variations in human subjects with high and low risks for obstructive sleep apnea (OSA). The platform was found to be highly selective to cortisol with minimal interference from unspecific adsorptions.

## Results and Discussion

**Figure 1:**
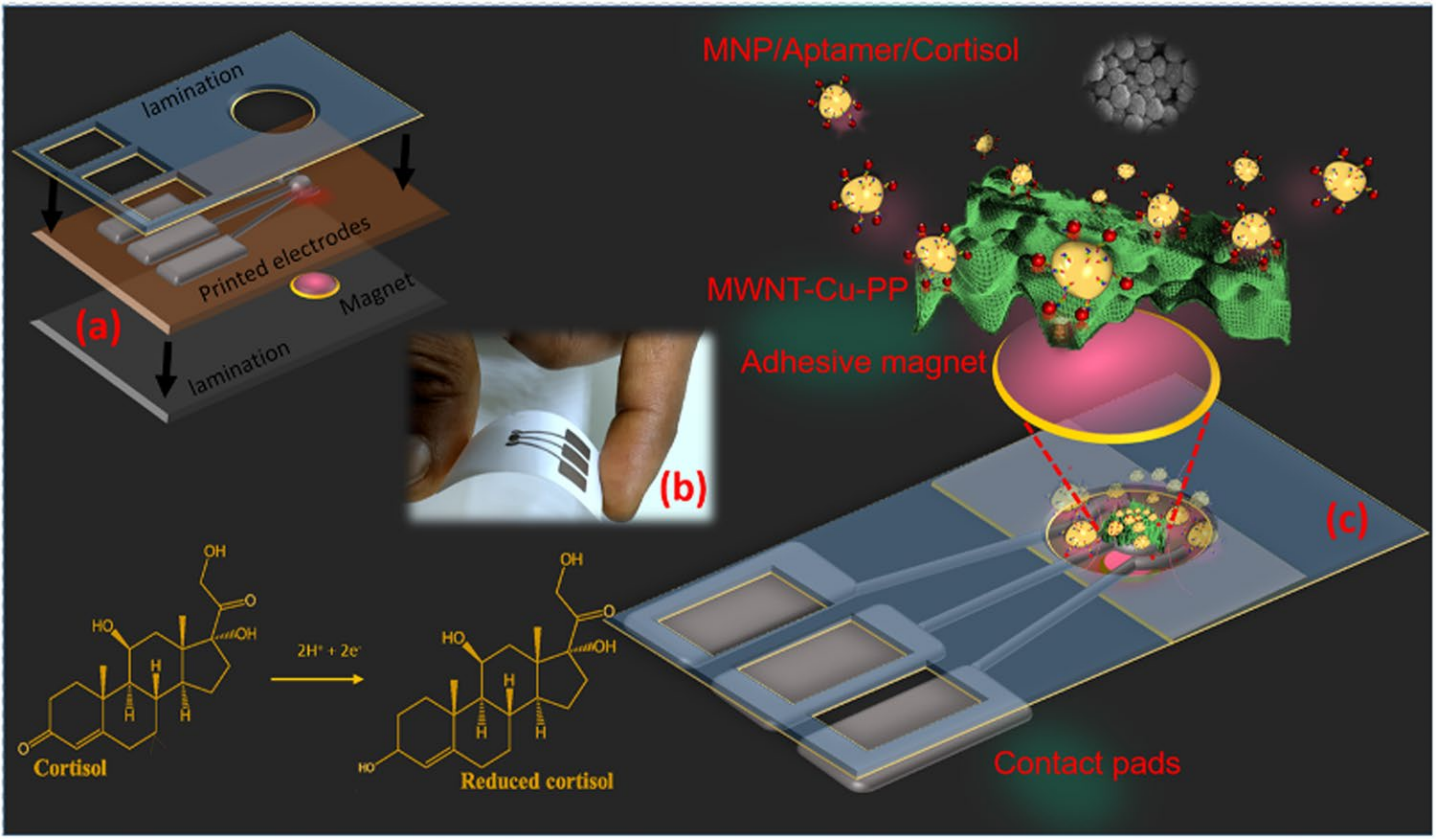
(**a**) Schematic of the disposable printed sensor which is enclosed in a plastic lamination with openings for contact pads and sensing area. A magnetic disc (r = 3 mm; t = 5 mm) is aligned and laminated at the back of the working electrode of the sensor (**b**) photograph of the sensor (**c**) The MNP/aptamer/cortisol complex is populated at the sensing electrode *via* magnetic enrichment where the reduction of cortisol occurs.

Sensors were printed using our custom carbon nanotube-Copper porphyrin ink (MWNT-Cu-PP) ink on a photo-paper substrate (Fig. 1). Sensor fabrication specifications are detailed in the materials and methods section. Extensive experimental and simulation studies were performed to establish the catalytic activity of Cu-PP in cortisol reduction. Catalytic activity of Cu-PP has been precisely analyzed using density functional theory (DFT) (Supplementary Materials ESI: S1†). Geometry optimization calculations were performed using Gaussian 09 DFT/B3LYP package.

**Figure 2:**
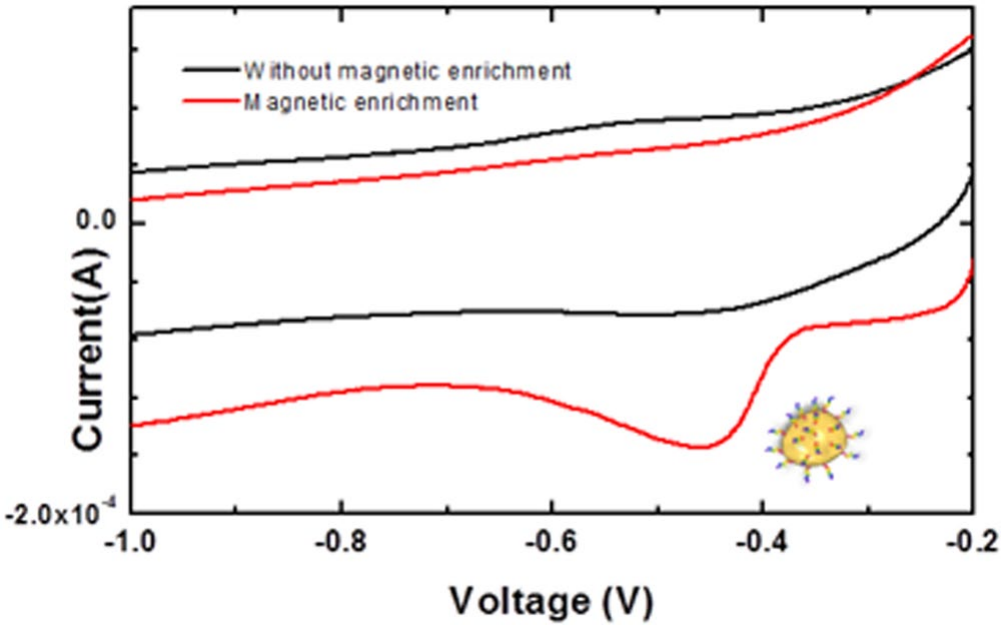
Cyclic voltammograms of 1 nM Cortisol at the MWNT-Cu-PP electrode in 0.1 M KCl, 10 mM phosphate buffer (pH 7.0) (1 nM) with and without MNP-AptC, aptamer tagged magnetic nanoparticle enrichment.

Based on the inherent specificity of an aptamer and MWNT-Cu-PP, a sensing platform was developed that is highly selective to cortisol. Magnetic nanoparticles (MNP), conjugated with a 61 bp cortisol aptamer (AptC), form MNP-AptC conjugates which are capable of selectively capturing cortisol in a solution. MNP-AptC is formed *via* biotin-streptavidin binding as a result of incubating streptavidin coated MNP with biotin modified AptC. Streptavidin- biotin interaction is biphasic as there are multiple biotin binding sites on a streptavidin molecule. The binding of streptavidin and biotin is extremely fast and a complete binding occurs in a matter of minutes as the streptavidin and biotin binding rate constant is in the range of 3.0 × 10^6^ − 4.5 × 10^7^ M^−1^ s^−1^ ^24^. Electrochemical measurements were performed using 50 μL solution. Measurements were done five minutes after introducing cortisol solution, treated with MNP-AptC conjugates, in the sensing area. MNP-AptC enriched the sensing surface as soon as it was introduced into the sensing area because magnetic nanoparticles were instantly pulled towards the surface of the sensing area by the magnetic disc that was sealed to the back of the sensor. The effect of the magnetic nanoparticles on cortisol enrichment was also notable. MWNT-Cu-PP electrodes, in conjugation with MNP-AptC, showed a marked reduction in current around −0.2 V, exclusively in the presence of cortisol (Fig. 2). Sensors enabled with a magnetic disc were found to yield ~350% higher currents than the sensors with no magnetic enrichment. The superlative catalytic response of MWNT-Cu-PP was also accredited to the edge planes of MWNT, which promoted high surface area, high electronic mobility and excellent electron transfer rate of composites^25–27^. Incorporation of MWNT enables fast electron transfer, increased electrode surface area and improved surface confined reactions. The edge plane of highly ordered pyrolytic graphite (HOPG) have unique open-end structure^28–30^. Researchers have demonstrated that the walls of the MWNT with large basal planes is highly conductive while its edges have high capacitance and electrocatalytic properties^30^. The heterogeneous nature of these MWNT, affects not only the electron transfer properties of the graphitic material, but it also has influence on the redox active molecules such as metalloporphyrins. Studies showed that the carbon nanotubes with large numbers of edge planes^28^ enhance the molecules’ redox activity by two orders of magnitude^30^.

**Figure 3:**
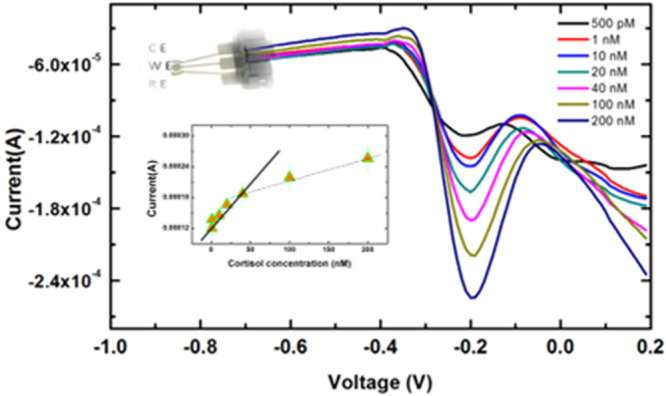
Differential pulse voltammograms at MWNT-Cu-PP-MNP-AptC for different cortisol concentrations in 0.1 M KCl, 10 mM phosphate buffer (pH 7.0) (**a**–**f**: 500 pM, 1, 10, 20, 40, 100, 200 nM) Inset: Cortisol concentration vs Output current.

Differential pulse voltammetry (DPV) characteristics showed a linear correlation with cortisol concentrations. The calibration curves have been derived from the average of three sensors outputs. The sensor recorded a proportional increase in current when cortisol concentrations were varied (Fig. 3). Although the limit of detection of the sensor was found to be 10 pM, a linear trend was observed in the 100 pM to 50 nM range **(**Fig. 3 inset). The linearity of the response was found to be lower, in the 50 to 200 nM range. This may be due to the MNP-AptC conjugate being unable to bind more cortisol.

### Selectivity

**Figure 4:**
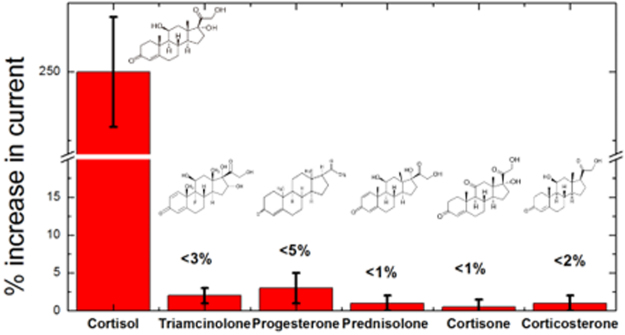
Percentage change in current response when Cortisol (1 nM) was detected in the presence of five closely related steroids at a tenfold high concentration (**a**) Triamcinolone Cortisone (**b**) Progesterone (**c**) Prednisolone (**d**) Cortisone (**e**) Corticosterone.

A series of tests were performed to ensure selectivity of our cortisol sensing platform to cortisol against the backdrop of five structurally similar steroids. Negative control experiments were performed with triamcinolone, progesterone, prednisolone, cortisone and corticosterone. As shown in Fig. 4, 1 nM of cortisol induced a significant electrochemical response, while all the other steroids generated negligible responses, even at a concentration tenfold higher than cortisol. These results indicated that our sensing system exhibits an excellent selectivity for cortisol and proved that co-existence of four closely related nonspecific steroids does not interfere with cortisol detection. Triamcinolone and progesterone recorded ~3% and ~5% change in reduction currents, respectively, while the responses of corticosterone and cortisone were less than 1% (Fig. 4, Fig. S6). In comparison, cortisol introduction recorded a ~250% increase in current. This considerable electrochemical activity of cortisol was a result of the operational synergy between aptamer probes and subsequent reduction at the MWNT-Cu-PP surface. Hence, we infer that signals from the electrochemical reduction of cortisol occur only for molecules that are specifically bound to the aptamer, whereas other closely related steroids that cannot bind to the aptamers are incapable of electron transfer to the MWNT-Cu-PP electrode surface.

**Figure 5:**
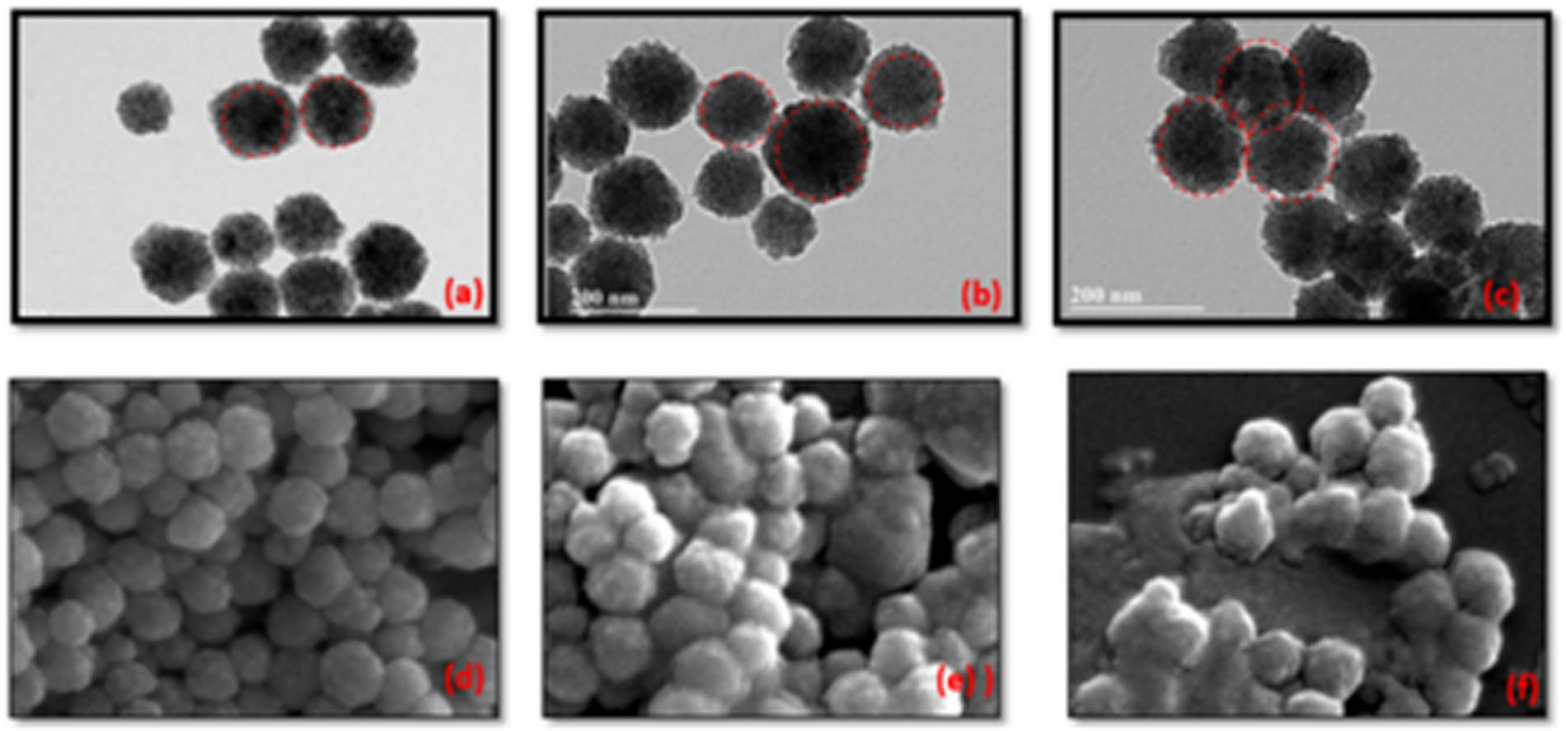
Nanoparticle aggregation with aptamer loading. (**a**–**c**): TEM images showing the aggregation of nanoparticles at >1 nM initial DNA concentration. Aptamer conjugation is proportional to the initial Aptamer concentrations- 1 nM, 10 nM and 40 nM respectively (**d**–**f**) SEM micrographs depicting the aggregation of nanoparticles at various aptamer concentrations.

The response was also found to be a strong function of the amount of aptamers per nanoparticle. The number of aptamers loaded on a nanoparticle can be controlled by changing the initial concentration of aptamers in the incubation solution. The average AptC density on MNP was characterized using florescent microscopy and ultraviolet spectroscopy (ESI: S1†). In order to probe the sensitivity of the assay to aptamer loading, experiments were conducted with three sets of MNP-AptC(i) conjugates were *where i* = 1, 10, 40 *nm*, the initial concentration of the aptamers. The relationship between initial aptamer concentration and aptamer loading was investigated using florescent imaging of FAM tagged biotin modified AptC. The florescence intensity from the MNP-AptC-FAM was found to be proportional to the number of aptamers loaded on the nanoparticles which was in turn a function of the initial concentration of aptamers. MNP-AptC(40) conjugate, formed using 40 nM of aptamer stock concentration, loaded 2.1 times more aptamers than MNP-AptC(1), nanoparticles loaded using a 1 nM stock (ESI: S1†, Fig. S4). Sensitivity and limit of detection (LOD) was also influenced by MNP-AptC(i). Measurements using MNP-AptC(1) were found to have a LOD of 10 pM. However, we observed that MNP-sensitivity was reduced to 30 pM when MNP-AptC(10) was used for sensing, although it had 35% more aptamer density than AptC(1). AptC(40) also recorded an LOD of 30 pM (ESI: S5, Fig. S5d†). Hence, a heavily loaded aptamer was found to capture less cortisol. This was due to a) high stearic hindrance offered by the densely packed immobilized aptamers or b) nanoparticle aggregation that resulted in lesser captured cortisol molecules in the vicinity of MWNT-Cu-PP surface or a combined effect of both (a) and (b). Scanning electron microscopy (SEM) and transmission electron microscopy (TEM) micrographs (Fig. 5a–f) of aptamer loaded nanoparticles confirmed considerable nanoparticle aggregation for aptamer loading beyond 7 × 10^12^ molecules/cm^2^. TEM (Fig. 5e–f) pictures revealed that particle aggregation is irreversible even under ultrasonic agitation. For our sensing platform we performed all the experiments using MNP-AptC(1) which generated a LOD of 10 pM which is appropriate for salivary cortisol analysis.

### Salivary cortisol measurements

Saliva contains 90% of the free cortisol^31^. Saliva samples, stored at −20 °C, were thawed at room temperature before experiments. Detection of cortisol levels in saliva is known to be influenced by digestive enzymes, neutral lipids and other proteins. We observed that untreated saliva samples, when introduced on the sensor surface, formed a froth-like precipitate. This may be due to proteins and other organic matter in the subject’s saliva. The extent of froth formation varied between samples. Hence, a short pretreatment was adopted to counter this problem, where the saliva samples were diluted in the reagent buffer (1:1) containing aptamer tagged magnetic nanoparticles. Following a short incubation for 60 seconds the solution was introduced into the sensing area of the device for electrochemical analysis. The sensing platform was used to measure cortisol levels in saliva samples collected from a pilot sample of subjects with high and low risks for Obstructive sleep apnea (OSA), a condition that can disrupt homeostatic mechanisms leading to hypothalamic-pituitary adrenal axis alterations and cortisol level dysregulation^32^. Polysomnography is the current gold standard to diagnose OSA, but it is expensive, time-consuming, and not ecologically valid^33^. Hence, a rapid way of distinguishing cortisol levels in individuals has good diagnostic value for identifying individuals with disrupted versus normal homeostatic mechanisms. Sensors showed consistent results with standard cortisol solutions. However, we observed a variation in the sensor output when saliva samples were introduced. This nonconformity is attributed to the variation in the amount of protein content and other organic/inorganic molecules in saliva. It has been reported that protein content in a sample of saliva can vary from 70–950 μg/mL even for the same subject^34^. Our initial studies also suggested that salivary cortisol estimation based on a standard calibration curve can lead to erroneous results. Hence, a standard addition assay was adopted for enhanced accuracy (Fig. S7†). Current response vs cortisol concentration were plotted for each reading and then extrapolated to derive the value of an unknown concentration. We found out a very good agreement between extrapolated values and the response of the unknown were in very good agreement.

**Figure 6:**
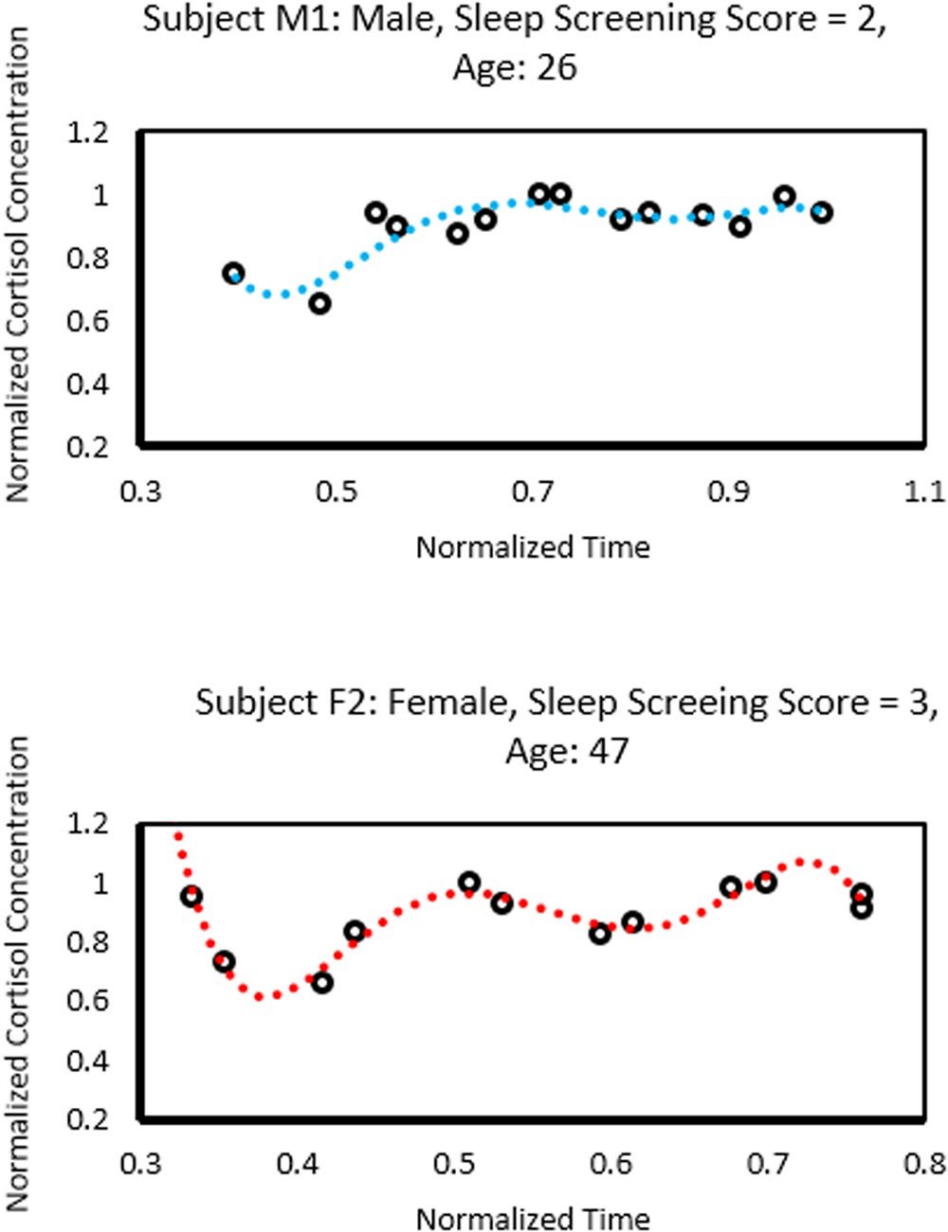
Salivary cortisol variations displaying variation amongst samples which were consistent with cortisol ultradian cycling. Figure 6a shows data from a male subject with low-risk of obstructive sleep apnea. Figure 6b shows data from a female subject with high-risk of obstructive sleep apnea.

Standard addition method was carried out by spiking known concentrations of cortisol in a saliva sample, and plotting a linear relationship. Enzyme Linked Immunosorbent Assay (ELISA) was used as the standard measurement. DPV measurements from the sensor were verified against ELISA using a series of standard addition method experiments to account for nonspecific biosignals. A good correlation between the electrochemical measurements and ELISA results was observed (ESI: Table S1). The cortisol concentrations obtained through the electrochemical method were slightly lower than the ELISA results, indicating possible electrochemical interference from saliva. The average cortisol signal recovered from the measurement is also listed in Table S1. The pilot sample consisted of five subjects, three women with high risk for OSA and two men with low risk for OSA. We noticed that there was a positive trend between increased age and risk for OSA, which was consistent with the literature^35^. Cortisol levels in the saliva samples of all subjects showed variance in cortisol levels which were consistent with ultradian cycling throughout the day. Normalization was done relative to the total time taken to recover the samples, which was on average 12 to 14 hours. Subjects with low risk for OSA showed less variance in normalized cortisol levels (Fig. 6a) compared to subjects with high risk of OSA (Fig. 6b). Based on these pilot data (Table 1), the sample size needed to detect differences in mean standard error of averaged normalized cortisol between women and men with a power of 80% is 16, assuming a significance level of 5%. Recovery values, which indicate the signal interference, were found to be ~89%. Signal loss from the sensing platform was eliminated during calibration by accounting for the signal interference. The standard addition assay confirms that the proposed aptamer-Cu-PP platform can be used for sensitive and rapid detection of cortisol. The sensor is not yet ready for point-of-care testing. With a customized electrochemical reader, the sensing platform can be used for point-of-care detection in future.

**Table 1:** Data and statistics for pilot sample showing sex, age, sleep screening (SS) score, and cortisol results; * indicate calculations using Wilcoxon Rank Sum Tests.

Pilot Sample	Age (years)	Sleep Screening Score	Averaged Normalized Cortisol			Mean Standard Error of Averaged Normalized Cortisol		
	Mean (Standard Deviation)			Minimum, Maximum	P-value*	Mean (Standard Deviation)	Mini mum, Maximum	P-value*
Women (n = 3)	56 (6)	3 (0)	0.878 (0.009)	0.869, 0.887	0.083	0.013 (0.005)	0.009, 0.018	0.083
Men (n = 3)	25 (1)	2 (0)	0.907 (0.006)	0.903, 0.911		0.007 (0.002)	0.005, 0.018	

## Materials and Methods

All experiments were performed using analytical grade reagents from Sigma-Aldrich. Cortisol Aptamer (AptC), a 61 bp aptamer functionalized with a biotin at the 5′ terminal position was obtained (IDT-DNA, Coralville, IA) 5′-biotin-AG CAG CAC AGA GGT CAGATG CAA ACC ACA CCT GAG TGG TTAGCG TAT GTC ATT TAC GGACC. FAM-modified aptamers were used for florescent studies (5′-biotin-AG CAG CAC AGA GGT CAGATG CAA ACC ACA CCT GAG TGG TTAGCG TAT GTC ATT TAC GGACC-FAM-3′)^36^. Streptavidin coated magnetic nanoparticles (100 nm, Spherotech, Inc) are suspended in phosphate buffer, pH 7.4 with 0.02% sodium azide. Cortisol ELISA Kit (Saliva) - Salimetrics Assays, were used for salivary cortisol analysis. ELISA was performed on a 96-well plate with respect to six known standard concentrations (0.00003, 0.03, 0.06, 0.2, 0.6, 1.5, and 4 μg/mL). JEOL SEM 6330 F Field Emission Scanning Electron Microscopy (Compositional and Topographic) equipped with Energy Dispersive Spectroscopy (EDS) was used for SEM characterization. The TEM was performed to study the particle distribution using Jeol, model JEM 200 CX.

### Aptamer loading and quantification

For aptamer loading on MNP, 1 nM, 10 nM and 40 nM stock solutions were added to 100 μL of 2.5% w/v magnetic nanoparticles in 50 mM Tris-HCl, 250 mM NaCl, 10 mM MgCl2, adjusted to pH 7.4 at 25 °C for one hour. Nanoparticles were washed using 20 mM Tris-HCl/0.1 M NaCl/5 mM MgCl2/1.0% (v/v) Tween 20 (pH 7.5). Reagent buffer used for electrochemical experiments: 0.1 M KCl, 10 mM phosphate buffer (pH 7.0).

The quantitative determination of aptamer loading was obtained by measuring the initial aptamer concentration before and after immobilization by correcting for the dilution factor. 100 μL of 2.5% w/v of Streptavidin coated Magnetic particles were separately added to 1, 10 and 40 nM biotin modified aptamer stock solutions in 50 mM Tris-HCl, 250 mM NaCl, 10 mM MgCl_2_, adjusted to pH 7.4 at 25 °C for one hour. Magnetic nanoparticles were collected by using a permanent magnet placed at the bottom of the tube. The supernatant was withdrawn, and its absorption spectrum was recorded at A260 as the peak at 260 nm corresponds to the DNA absorption (ESI: S5, Fig. S5a–c†). The number of aptamers loaded on the nanoparticles (AptC) was determined by finding out the difference in the aptamer concentration before and after MNP introduction.

$$[{\rm{Aptamer}}/{\rm{mL}}]=[{\rm{conc}}\,{\rm{NP}}]({\rm{nanoparticles}}/{\rm{mL}})\times {\rm{loading}}\,{\rm{capacity}}\,({\rm{oligos}}/{\rm{particle}})$$

### Sensor fabrication

Electrodes were printed onto a photo-paper substrate using aqueous ink formed by using a combination of solvents under vigorous stirring. The silver nanoparticles are prepared by the reduction of AgNO_3_ in Ethylene glycol solution in the presence of polyvinyl pyrrolidone (PVP). A 1:1.8 ratio of AgNO_3_ and PVP were maintained in ethylene glycol (EG). Then, the mixture was heated up to 120 °C in an oil bath with vigorous magnetic stirring for 30 min. Ink was composed of silver nanoparticles with particle diameter less than 10 nm. The mixture solution was also ultrasonically agitated for three minutes. The obtained products were centrifuged at 12,000 rpm for 5 min and washed five times in ethanol. Subsequently, silver nanoparticles were centrifuged and then dissolved in ethanol and stirred for 15 min. MWNT-Cu-PP conjugate were initially prepared by mixing 5 mg of MWNT with 0.5 mmol of 5,10,15,20-Tetraphenyl-21H,23H-porphine copper(II) (Cu-PP) in 10 ml Dimethylformamide (DMF). This conjugate was later mixed with silver nanoparticle-ethanol solution and sonicated for 30 minutes to form the MWNT-Cu-PP ink. The ink containing 20 wt% silver has a viscosity of ~9.5 cP and a surface tension of ~36 mN m−1, meeting the requirements inkjet printer(MFC-J680DW). Kodak premium photo-paper, gloss finish, 8.5 mil thickness, was used as the photo-paper substrate.

These printed electrodes enhanced with carbon nanotube-Copper porphyrin ink were used in conjunction with aptamer-tagged magnetic nanoparticles to detect salivary cortisol variations. A magnetic disc, r = 5 mm (Uline, WI) was laminated to the back of the sensing area of the inkjet printed sensor (Fig. 1). The whole assembly was sealed in a 2 × 1 cm^2^ lamination (Thickness: 250 μm).

### Electrochemical measurements

Cyclic voltammetry and differential pulse voltammetry were performed using a CHI 1220 Electrochemical Analyzer (CH Instruments). The potential range in cyclic voltammetry was from −1.6 to −0.2 V with scan rate 50 mV/s. The electrodes tested were printed sensors in 50 µL of 0.1 M KCl, 10 mM phosphate buffer (pH 7.0). Measurements were done after five minutes of sample introduction. Similarly, differential pulse voltammetry was conducted with a pulse amplitude of 50 mV, pulse width of 0.05 s, and pulse period of 0.5 s. The electrodes and conditions were similar to that of cyclic voltammetry.

### Saliva collection

Adult subjects were recruited according to protocol #16-259 approved by the University of Missouri-Kansas City Institutional Review Board, their rights were protected and all provided written informed consent to participate. At the initial study visit, subjects completed medical history and sleep screening forms^37^. Those with a major systemic disease or psychiatric disorder, cancer, or xerostomia were excluded along with anyone who was pregnant or who smoked cigarettes. Subjects who qualified were classified according to their responses to Stop-Bang-questions, a list of 8 questions in the sleep screening form, where ≥3 “yes” responses defined those with high risk and ≤2 “yes” responses defined those with low risk for OSA(ESI: S3†). Stop-Bang-Score is defined by the number of positive responses. Subjects were trained to collect saliva samples every 2 hours for 2 days from awakening until sleeping times, to avoid eating or drinking fluids except water 30 minutes before sampling, complete a diary to record sequential sample numbers and timing of sample collection for each day, and store samples in a freezer until their second visit. Specifically, to collect and store samples, each subject placed one of the sterile cotton pellets provided under her/his tongue for about 5 seconds, recorded the time of day in a diary form, then removed the saliva-soaked pellet and placed it directly into the sterile plastic tube provided that was labelled with the appropriate day and sample number (Fig. S2). After securing the attached cap on the tube, the tube with sample was deposited in a plastic zip-top bag labelled with the appropriate day and the bag was stored in a freezer. This process was repeated every two hours until retiring to sleep. At the second visit the subjects completed their participation by delivering their diary forms and frozen samples to investigators.

## Conclusion

A point-of-care sensing platform for salivary cortisol detection has been reported. We developed a macrocyclic catalyst ink based printed electrodes capable of electrochemically reducing salivary cortisol captured by aptamer functionalized magnetic nanoparticles. The paper-plastic sensor consists of a thin magnet disc to populate the magnetic nanoparticle bound cortisol at the sensing electrode area. High selectivity was observed to salivary cortisol against a background of four closely related steroids (triamcinolone, progesterone, cortisone and corticosterone). The potential of the sensing platform was demonstrated by detecting the salivary cortisol variations of five human subjects with high and low risks for obstructive sleep apnea (OSA). The sensing platform detected the variations typical of ultradian cycling and detected the increased variance for those with high risk for OSA.

## Electronic supplementary material

Supplementary information
