# Supplementary material for: Disposable aptamer-sensor aided by magnetic nanoparticle enrichment for detection of salivary cortisol variations in obstructive sleep apnea patients
Source: Sci Rep. 2017 Dec 21;7:17992. doi: 10.1038/s41598-017-17835-8 (PMC5740141; doi:10.1038/s41598-017-17835-8)
Supplement: Supplementary file 1 — Supplementary information [file 41598_2017_17835_MOESM1_ESM.pdf]

SUPPLEMENTARY MATERIAL

Disposable aptamer-sensor aided by magnetic nanoparticle enrichment for detection of salivary cortisol variations in obstructive sleep apnea patients

Renny Edwin Fernandez[1], Yogeswaran Umasankar[2], Pandiaraj Manickam[1], Jeffrey C. Nickel[3], Laura R. Iwasaki[3], Burt K. Kawamoto[4], Kristen C. Todoki[4], JoAnna M. Scott[5], Shekhar Bhansali[1]

[1]Bio-MEMS and Microsystems Laboratory, Department of Electrical and Computer Engineering, Florida International University, Miami, Florida

[2]Biomolecular Sciences Institute, Florida International University, Miami, Florida.

[3]School of Dentistry, Departments of Orthodontics and Dentofacial Orthopedics and Oral and Craniofacial Sciences, University of Missouri-Kansas City, Kansas City, Missouri

[4]School of Dentistry, University of Missouri-Kansas City, Kansas City, Missouri

[5]School of Dentistry, Department of Oral and Craniofacial Sciences, University of Missouri-Kansas City, Kansas City, Missouri

S1: The proposed energy profile of Cu-PP catalysis

Energy profile and transition state structure of three metalloporphyrins(M-PP), namely Cu-PP, Ruthenium metalloporphyrin (Ru-PP) and Nickel metalloporphyrin (Ni-PP) were studied to analyze the catalytic property of these metal-porphyrin conjugates. Catalytic activity of these molecules to cortisol reduction was modelled precisely using density functional theory (DFT). Geometry optimization calculations were performed using Gaussian 09 DFT/B3LYP package. Initially, the oxygen atom (at C_3 position) of the cortisol molecule interacts with M-PP *via* its central metal ion. The graphical representation of the complex formation using Cu-PP-cortisol system is shown in Figure S1.

Cortisol has five important oxygen containing binding sites (hydroxyl or ketone group) at C_3, C_{11}, C_{17}, C_{20}, and C_{21} positions. Out of the five significant binding sites of cortisol, there has been a lot of focus on the reduction of the C-3 carbonyl group. (Goyal et al., 2010). Thus, the most probable site for reduction in cortisol is at position 3 where the keto group undergoes reduction in 2^{e^-}, $2H^+$ process to give a hydroxyl group. So all the simulation studies of cortisol were performed at C-3 position. The optimized Cu-O distance between Cu atom of CuTPP and O atom (C_3) of cortisol was 2.74Å (Fig. S1). Subsequently during the transition state, the oxygen atom of cortisol moves towards the Cu catalyst to form a Cu-O bond. **Fig. S1** shows the energy profile of cortisol binding with CuTPP. The transition state (TS, the highest energy point along the reaction pathway) was calculated for the reaction. In the triplet ground state CuTPP has a strong binding

energy (−15.15 kcal/mol) with cortisol. Binding energies were computed as the difference between the energy of the complex and the energy of each molecule.

Binding energies were computed as the difference between the energy of the complex and the energy of each molecule. Among all the M-PPs, Cu-PP was found to have the highest activity for cortisol. In the triplet ground state, Cu-PP had the strongest binding energy (−15.15 kcal/mol), compared to Ru (-14.12 kcal/mol) and Ni porphyrin (-13.38 kcal/mol). For the optimized structures, the calculated metal-oxygen (M-O) distances are 2.71Å, 2.84Å, and 2.74Å for Ni-O, Ru-O, and Cu-O respectively (**Figure S1)**.

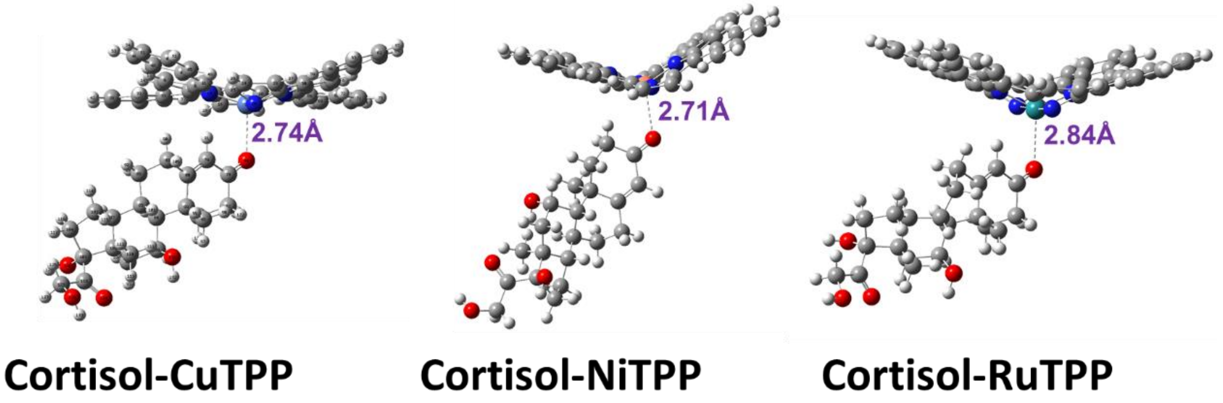

Figure S1a: Calculated structures of MTPP-cortisol complex at transition state with bond lengths in angstroms.

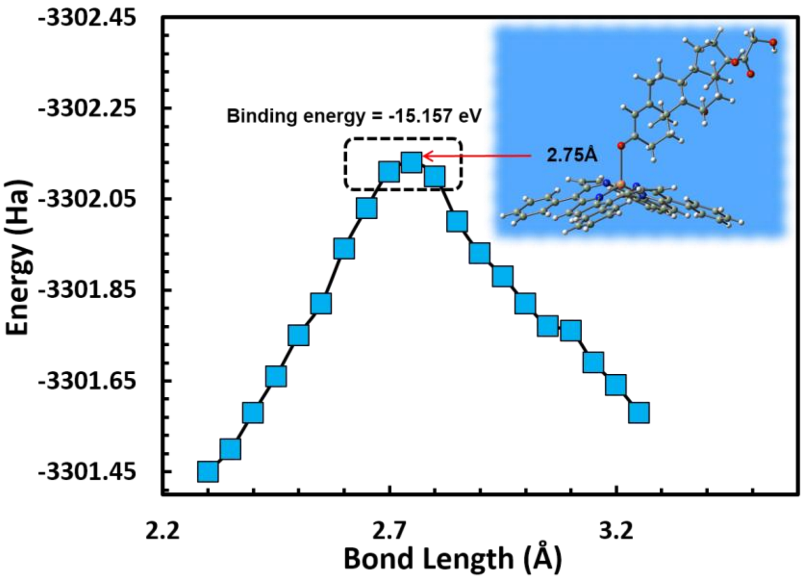

Fig. S1b. The proposed energy profile and transition state structure for CuTPP catalyzed reduction of cortisol. The inset shows the cortisol-CuTPP complex at trastion state. Orange, red, blue, gray, and white balls represents Copper, oxygen, nitrogen, carbon, and hydrogen respectively. ΔE is binding energy (ΔE = E (CuTPP – cortisol complex) − E (CuTPP)−E (Monomer).

Experimentally, cortisol in presence of Cu-PP, were observed to have a reduction peak (Ep_c) at -0.45 V which is attributed to the the reduction of cortisol (Ip_c of $1.8x10^{-4}$ A). However, no reduction peaks we observed at Ep_c -0.45 V in the absence of Cu-PP (Fig. S1c).

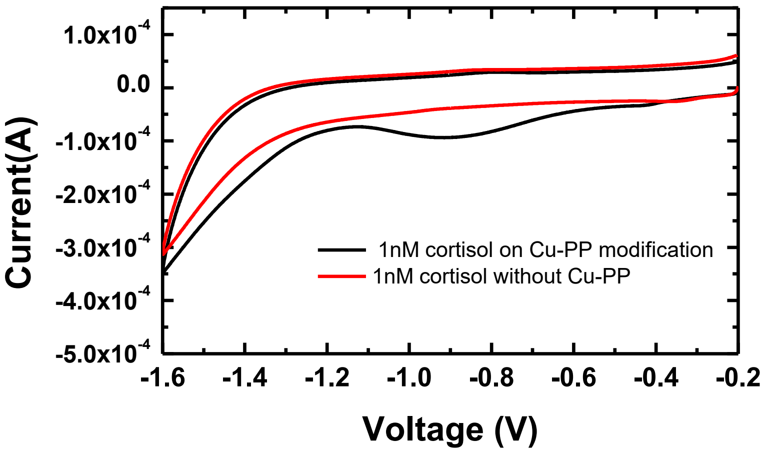

Fig. S1c. Cyclic voltammograms of 1 nM Cortisol at the printed electrode in 0.1 M KCl, 10 mM phosphate buffer (pH 7.0) with and without Cu-PP

* Goyal, R.N., Chatterjee, S., Rana, A.R.S., 2010. Talanta 83, 149–155.

S2: Saliva sample collection

Fig. S2 shows how subjects were to place a cotton pellet sublingual for 5 seconds or until the pellet was thoroughly moist. Subjects were then asked to place the moist cotton pellet in the respective labeled tube, and stored in a freezer until time of collection (visit #2). Subject participants marked the time that each salivary sample were taken along with any activities occurring during that time in a study diary for two sample collection days (**Fig. S3**). Study diary was used to record time of samples taken, and allowed us to remark on any deviations that may have occurred in salivary cortisol levels.

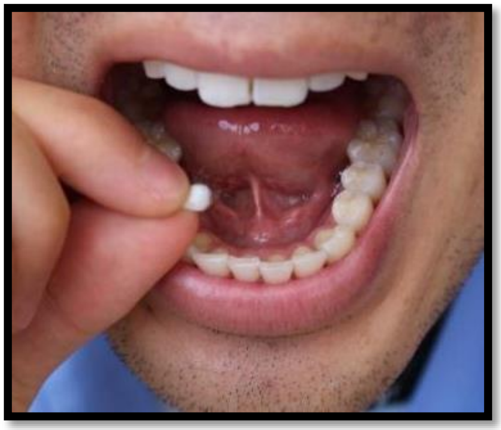

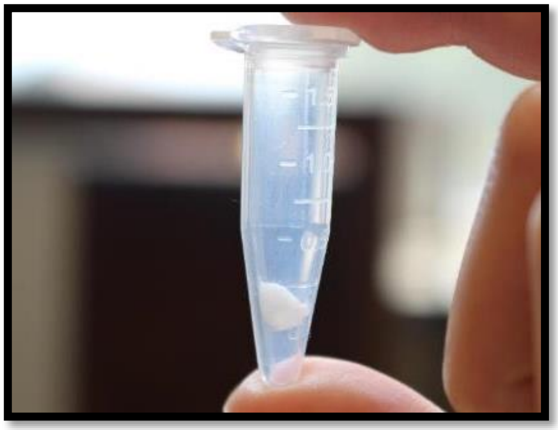

Fig. S2: Saliva sample a: collection with cotton pellet and b: storage in sealed tube

S3: Sleep Screening Questionnaires**

Please answer the questions below to help us assess for possible sleep apnea, a condition in which your breathing pauses or stops for periods of time while you sleep. Sleep apnea can increase your risk for many health conditions. It can also increase your risk for breathing problems after surgery.

Subject #____ Date ______________________

Age (years) ___________Height (feet, inches) ______________________ Weight (pounds) __________________

	Yes	No
Have you ever been diagnosed with obstructive sleep apnea (OSA)?	☐	☐
Are you currently being treated for OSA?	☐	☐
Are you aware of a family history of OSA?	☐	☐
Are you aware of clenching or grinding your teeth at night?	☐	☐

ESS: Epworth Sleepiness Scale

How likely are you to doze off or fall asleep in the following situations, in contrast to just feeling tired?

0 = I would never doze
1 = I have a slight chance of dozing
2 = I have a moderate chance of dozing
3 = I have a high chance of dozing

Situation	Chance of Dozing
1. Sitting and reading	________
2. Watching TV	________
3. Sitting inactive in a public place (e.g. a theatre or a meeting)	________
4. As a passenger in a car for an hour without a break	________
5. Lying down to rest in the afternoon when circumstances permit	________
6. Sitting and talking to someone	________
7. Sitting quietly in a lunch without alcohol	________
8. In a car while stopped for a few minutes in traffic	________

STOP - BANG

			Yes	No
1.	**S**nore	Do you snore loudly? (Louder than talking or loud enough to be heard behind a closed door?	☐	☐
2.	**T**ired	Do you often feel tired, fatigued or sleepy during daytime?	☐	☐
3.	**O**bstruction	Has anyone observed you stop breathing during your sleep?	☐	☐
4.	**P**ressure	Do you have or are you being treated for high blood pressure?	☐	☐
5.	**B**MI	Is your body mass index greater than 28?	☐	☐
6.	**A**ge	Are you 50 years old or older?	☐	☐
7.	**N**eck	Are you a male with a neck circumference greater than 17 inches, or a female with a neck circumference greater than 16 inches?	☐	☐
8.	**G**ender	Are you a male?	☐	☐

____

(**Chung, F., Yegneswaran, B., Liao, P., Chung, S. A., Vairavanathan, S… & Shapiro, C. M. (2008). *The Journal of the American Society of Anesthesiologists*, 108(5), 812-821)

**Day**	**Time**	**Notes: What activities were you doing? How do you feel?**
Sample 1		
Sample 2		
Sample 3		
Sample 4		
Sample 5		
Sample 6		
Sample 7		
Sample 8		

Fig. S3: Subject study diary

S4: <u>Aptamer loading</u>:

A series of initial studies revealed that the number of aptamers on nanoparticles were

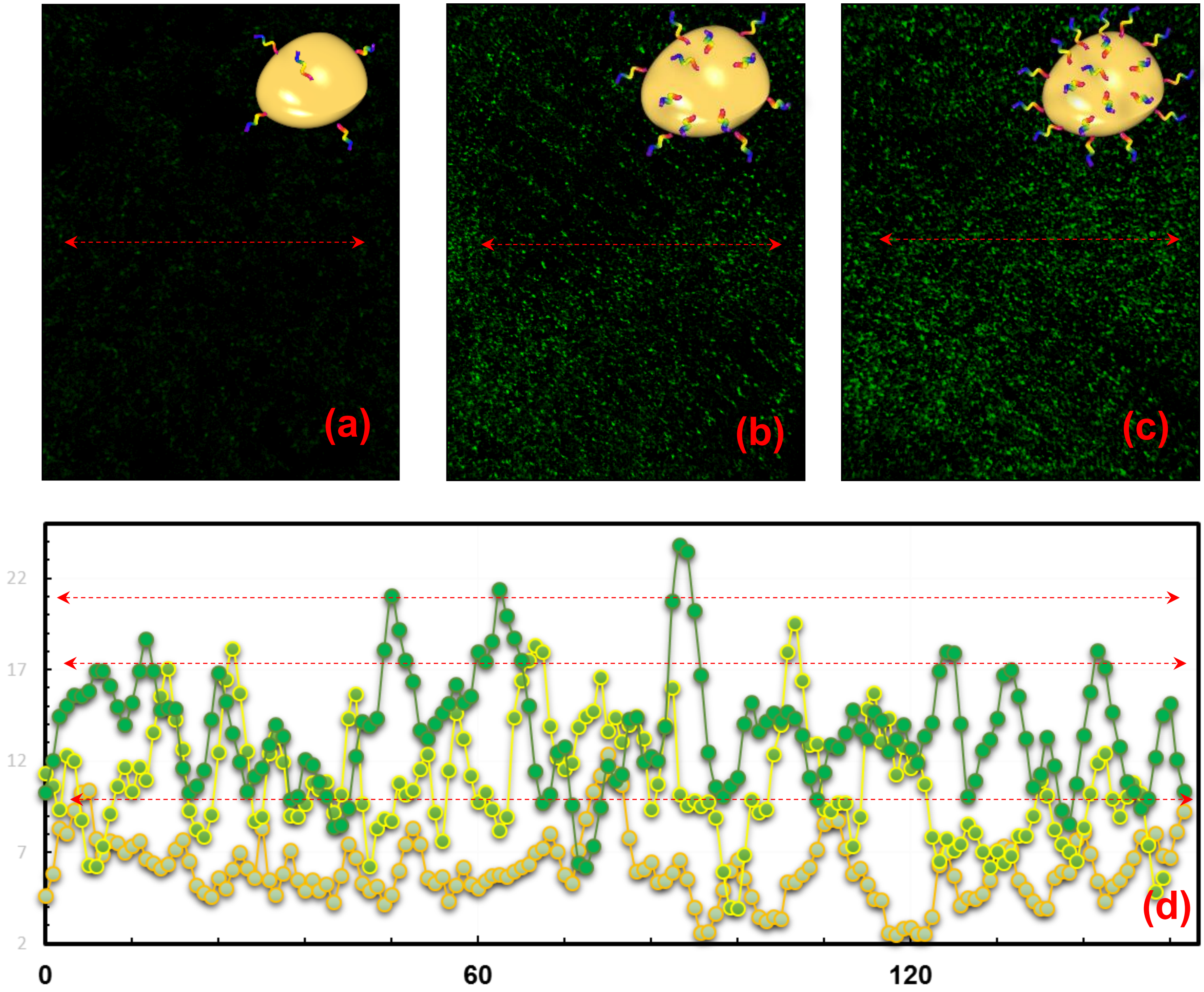

Fig. S4: Florescent tagged aptamers on Fe2O3 nanoparticles. (a-c): Fluorescence microscopy images of aptamer conjugation is proportional to the initial aptamer concentrations- 20 nM, 50 nM and 100 nM respectively d) Florescent intensity plot showing the variation in florescence intensity with aptamer loading

found to influence the sensitivity of the assay [1-3]. To estimate the influence of aptamer loading on MNP, FAM tagged biotin modified AptC were loaded on magnetic

nanoparticles. 5'-biotin-AG CAG CAC AGA GGT CAGATG CAA ACC ACA CCT GAG TGG TTAGCG TAT GTC ATT TAC GGACC-FAM-3'. The florescence intensity from the MNP-AptC-FAM was used to determine aptamer loading on nanoparticles. We observed a strong correlation between the amount of aptamers loaded on the nanoparticles and the initial concentration of S-MNp in the reagent buffer. Fluorescence micrographs (Fig **S4**) reveal that the nanoparticles immobilized using 1 nM of aptamers loaded ~ 85 % more aptamers than the nanoparticles loaded using a 100 pM stock of aptamers. Comparative studies also confirmed that the lower detection limit as well as the linear range was also a function of aptamer loading. Fluorescence microscopy images were taken using a Zeiss Axio Imager A1 fluorescence microscope with a Canon G6 digital camera attached. SEM and TEM images also confirmed that overloading nanoparticles with aptamers can lead to irreversible nanoparticle aggregation. Magnetic nanoparticles has been used successfully as solid phase support in immunoassays. Their ability to isolate various molecules in applications such as ribonucleic acid (RNA) purification, magnetic cell separation, magnetic particle enzyme immunoassay (EIA) and many other applications makes them beneficial to molecular and cellular isolations. The estimated oligonucleotide loading capacity of magnetic nanoparticles based on the diameter and concentration of nanoparticles is 7 x 10^{12} aptamers/cm^2 for a stock solution of 1 nM AptC.

1. Tan, Y., Shi, Y. S., Wu, X. D., Liang, H. Y., Gao, Y. B., Li, S. J., ... & Gao, T. M. (2013). DNA aptamers that target human glioblastoma multiforme cells overexpressing epidermal growth factor receptor variant III in vitro. *Acta Pharmacologica Sinica*, *34*(12), 1491.
2. Attarwala, H., & Amiji, M. (2012). Multi-compartmental nanoparticles-in-emulsion formulation for macrophage-specific anti-inflammatory gene delivery. *Pharmaceutical research*, *29*(6), 1637-1649.
3. Liao, K. T., Tsegaye, M., Chaurey, V., Chou, C. F., & Swami, N. S. (2012). Nano-constriction device for rapid protein preconcentration in physiological media through a balance of electrokinetic forces. *Electrophoresis*, *33*(13), 1958-1966.

S5: Aptamer loading:

The quantitative determination of aptamer loading was obtained by measuring the initial aptamer concentration before and after immobilization by correcting for the dilution factor. Aptamer concentrations were determined from A_{260} as the peak at 260 nm corresponds to the DNA absorption. The absorbance of a solution increases as attenuation of the beam increases. Absorbance is directly proportional to the path length and the concentration of the absorbing species.

(a) [Aptamer/mL] = [conc NP] (nanoparticles/mL) × loading capacity (oligos/ particle)

(b) Conversion to moles: (mol/ mL) = [Aptamer / mL] ÷ 6.022×10^{23} (Aptamer /mol)

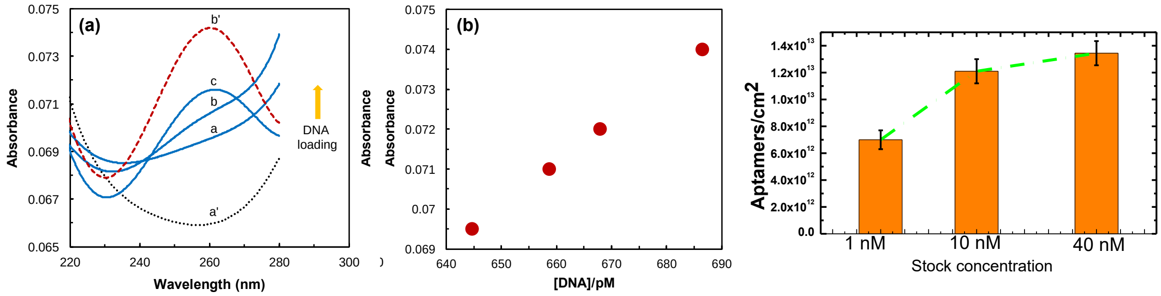

Fig S5: a) Absorbance spectroscopy signals of magnetic nanoparticles loaded with aptamers using 1, 10 and 40 nM stock solutions b) The UV absorbance intensity at 260 nm against different concentration of aptamer stock c) Initial stock concentration Vs average aptamer loading (molecules/cm^2).

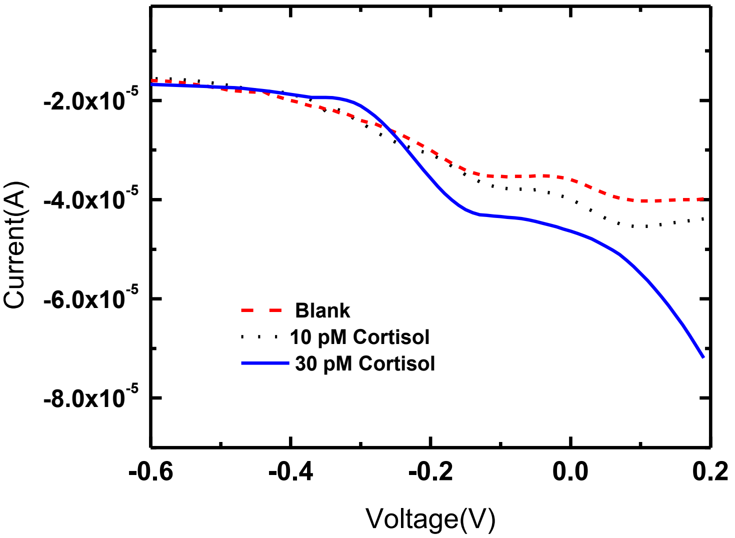

Fig S5: d) Differential pulse voltammograms at MWNT-Cu-PP-MNP-AptC for different 10 and 30 pM cortisol concentrations in 0.1 M KCl, 10 mM phosphate buffer (pH 7.0)

we observed that AptC (1) which had an average aptamer density of 7.5×10^{12} aptamers/cm^2 displayed a limit of detection(LOD) of 10 pM while AptC (10) which had 35% more aptamers than AptC (1) had a limit of detection of 30 pM (Fig. S5-d).

S6: Selectivity studies:

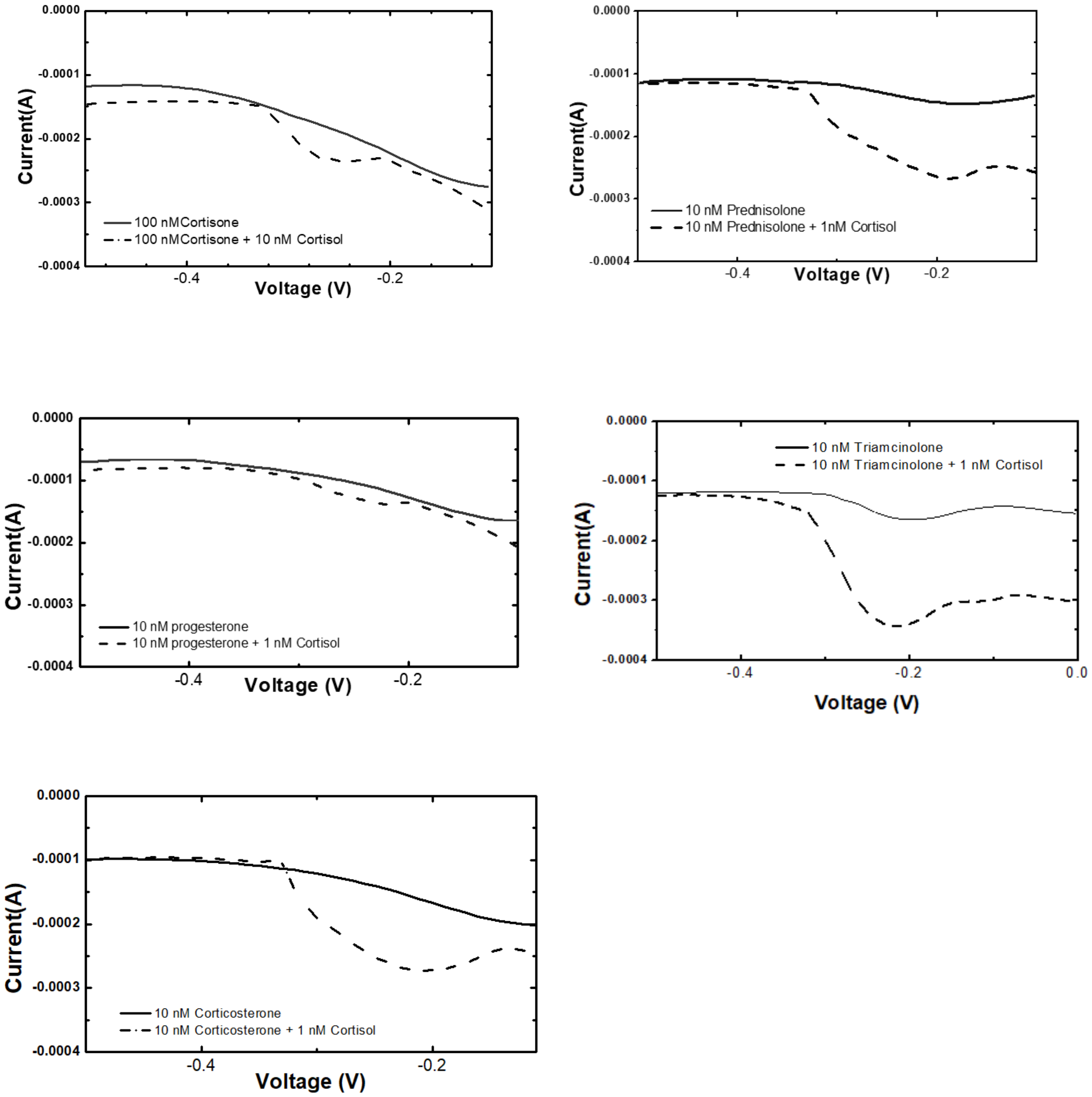

Fig S6: Current response of the sensor when Cortisol (1nM) was measured in the presence of four closely related steroids a) Cortisone (10 nM) b) Prednisolone c) Progesterone d) Triamcinolone e) Corticosterone

Sample number	Elisa/Added (nM)	Found (nM)	RSD (%)
1	4.9±0.2	5.2±0.5	7
2	3.4±0.4	3.1±0.2	31
3	5.1±0.6	4.9±0.4	37
4	4.2±0.1	3.4±0.3	5
5	5.1±0.5	5.1±0.6	0.7
6	4.9±0.4	4.5±0.4	8
1	3.8±0.3	4.2±0.2	21
2	3.3±0.2	4.1±0.1	3
3	4.6±0.1	4.4±0.3	6
4	4.6±0.4	4.1±0.2	3
5	5.1±0.5	5.3±0.5	1
6	4.8±0.2	4.4±0.1	7
7	4.6±0.2	5.1±0.5	4

Table S1. Real sample studies –cortisol: Comparison of cortisol measurements and ELISA in biosensor. Table shows the RSD values of electrochemical measurements of cortisol in saliva.

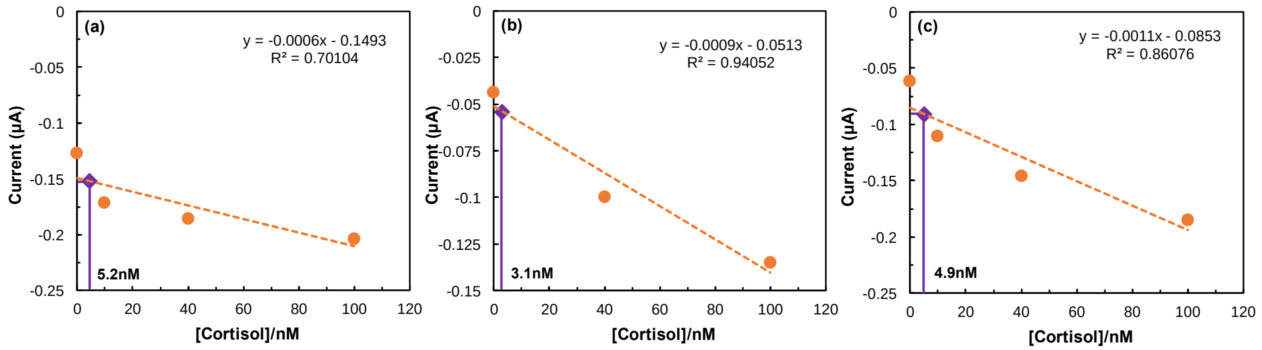

Fig. S7: Standard Addition plots for the determination of salivary Cortisol levels.
